# Immunomodulation and Anti-Inflammatory Effects of Garlic Compounds

**DOI:** 10.1155/2015/401630

**Published:** 2015-04-19

**Authors:** Rodrigo Arreola, Saray Quintero-Fabián, Rocío Ivette López-Roa, Enrique Octavio Flores-Gutiérrez, Juan Pablo Reyes-Grajeda, Lucrecia Carrera-Quintanar, Daniel Ortuño-Sahagún

**Affiliations:** ^1^Psychiatric Genetics Department, National Institute of Psychiatry, “Ramón de la Fuente”, Clinical Research Branch, Calzada México-Xochimilco 101, Colonia San Lorenzo Huipulco, Tlalpan, 14370 Mexico City, DF, Mexico; ^2^Unidad de Genética de la Nutrición, Instituto de Investigaciones Biomédicas, Universidad Nacional Autónoma de México, Instituto Nacional de Pediatría, Avendia del Iman No. 1, Cuarto Piso, 04530 Mexico, DF, Mexico; ^3^Departamento de Farmacobiología, CUCEI, Universidad de Guadalajara, Boulevard Marcelino García Barragán, No. 1421, Esq. Calzada Olímpica, 44430 Guadalajara, JAL, Mexico; ^4^National Institute of Psychiatry, “Ramón de la Fuente”, Clinical Research Branch, Calzada México-Xochimilco 101, Colonia San Lorenzo Huipulco, Tlalpan, 14370 Mexico City, DF, Mexico; ^5^Instituto Nacional de Medicina Genómica, Periférico Sur No. 4809, Colonia Arenal Tepepan, Delegación Tlalpan, 14610 México, DF, Mexico; ^6^Instituto de Investigación en Ciencias Biomédicas (IICB), CUCS, Universidad de Guadalajara, Sierra Mojada No. 950, Colonia Independencia, 44340 Guadalajara, JAL, Mexico

## Abstract

The benefits of garlic to health have been proclaimed for centuries; however, only recently have *Allium sativum* and its derivatives been proposed as promising candidates for maintaining the homeostasis of the immune system. The complex biochemistry of garlic makes it possible for variations in processing to yield different preparations with differences in final composition and compound proportion. In this review, we assess the most recent experimental results, which indicate that garlic appears to enhance the functioning of the immune system by stimulating certain cell types, such as macrophages, lymphocytes, natural killer (NK) cells, dendritic cells, and eosinophils, by mechanisms including modulation of cytokine secretion, immunoglobulin production, phagocytosis, and macrophage activation. Finally, because immune dysfunction plays an important role in the development and progress of several diseases, we critically examined immunoregulation by garlic extracts and compounds isolated, which can contribute to the treatment and prevention of pathologies such as obesity, metabolic syndrome, cardiovascular disorders, gastric ulcer, and even cancer. We concluded that *A. sativum* modulates cytokine secretion and that such modulation may provide a mechanism of action for many of their therapeutic effects.

## 1. Introduction

Plants of the genus* Allium* are known for their production of organosulfur compounds, which possess interesting biological and pharmacological properties. Among these, garlic (*Allium sativum*) is one of the most widely used ones. When extracted and isolated, these compounds exhibit a broad spectrum of beneficial effects against microbial infections as well as cardioprotective, anticancerigenic, and anti-inflammatory activity [1–5].

Preparations of garlic are mainly liquid (aqueous, oil, or solvent extracts) or solid (dried garlic powder and fresh cataplasm). These extractions can be based on water formulations, oils, or by using solvents as alcohols [6]. Composition of the extracts depends on the source of the garlic strain, age, storage conditions, and type of processing, and the effects of the extracts are influenced by the method of consumption [7]. Biological effects of different garlic preparations and extracts are summarized in Table 1.

The wide variety of effects that has been reported of garlic preparations and extracts with beneficial and useful properties may be due to their numerous compounds (organosulfur and others) contained in different concentrations, which is being a challenge to separate and identify compounds with potential beneficial properties on the human immune and cardiovascular systems [7]. A comprehensive classification of the different compound derived from garlic, as well as their biological effects reported, is actually in preparation and will be published elsewhere (Rodrigo-Arreola et al., in preparation). The presence and potency of garlic compounds vary with respect to mode of garlic preparation and extraction. Additionally, the proportion of these compounds is poorly controlled with the methods used to generate different garlic preparations, the main problem being reproducibility and validation of the real effects observed.

## 2. Main Organosulfur Compounds Purified from Garlic Preparations

The presence of garlic compounds varies with respect to mode of garlic preparation and extraction as follows: (1) fresh bulbs main compounds are S-allyl-L-cysteine sulfoxide (alliin) and *γ*-glutamyl cysteine derivatives; (2) in steam distilled oils, sulfide family compounds are the main compounds; (3) powder from crushed and dried garlic contains alliin and diallyl disulfide (DADS); (4) macerates (ground garlic) are enriched extractions with sulfide family compounds, dithiines, and (E–Z)-ajoene compounds, and (5) AGE (soaked, sliced, aged garlic extract in ethanol solution) contains S-allyl-L-cysteine (SAC) and S-allyl mercaptocysteine (SAMC) [40].

Garlic compounds can be divided in several groups or families of compounds. Among these families, we find *γ*-glutamyl cysteine derivatives, the primary precursor components of the alliin and allyl methyl cysteine (methiin) compound families [6, 41], that produce, by enzymatic action of alliinase (alliin lyase, EC: 4.4.1.4), the diallyl thiosulfinate (Allicin) and allyl methyl thiosulfinate (AM) compound families [41, 42], which are precursors of several organosulfur compound families (i.e., the ajoene and dithiin families) [8]. Additionally, garlic preparations contain nonorgan sulfured compounds, such as tetrahydro-beta-carbolines [43, 44], fructans, and glucose-linked *β*-D-fructofuranosyl [45], identified in AGE preparations [25].

## 3. Immunomodulatory Properties of* Allium sativum*

Immunomodulation is one of the main targets for synthetic drugs and chemicals. However, its high cost, anticipated toxicity, and adverse event effects render it undesirable for the patients. In contrast, the use of herbal plants as health promoters is gaining increasing attention in both consumers and scientific circles. In the literature, several plants have been listed that exhibit immunomodulatory actions, like modulation of cytokine secretion; phagocytosis promotion and macrophage activation; immunoglobulin production; allergic reactions and lymphocyte proliferation [46]. Recently, garlic has been suggested as a promising candidate for maintaining the homeostasis of the immune system. Several studies have been carried out in animal models to examine the effect of different garlic components and formulations on immunomodulatory activities (summarized in Table 2).

### 3.1. Modulation of Cytokine Secretion by Garlic Derivatives

Herbal medicines with immunomodulatory activity alter the immune function through the dynamic regulation of molecules such as cytokines and chemokines. Altering cytokine expression and targeting their receptors may offer therapeutic potential. Current pharmacological strategies include cytokine antagonist, agonist, inhibition, and stimulation models. However, in light of the adverse events experienced with cytokine-targeted therapy, it could be useful to consider the use of phytotherapy in the modulation of cytokine expression [47]. Recently, Quintero-Fabián et al. examined the effects of alliin in lipopolysaccharide- (LPS-) stimulated 3T3-L1 adipocytes. Incubation of cells for 24 h with 100 *μ*mol/L alliin prior to LPS (100 ng/mL) stimulation for 1 h prevented an increase in the expression of proinflammatory genes* IL-6*,* MCP-1*, and* Egr-1* and in the protein levels of IL-6 and MCP-1. Interestingly, the phosphorylation of ERK1/2, which is involved in LPS-induced inflammation in adipocytes, decreased following alliin treatment. Furthermore, gene expression profile by microarray evidences an upregulation of genes involved in immune response and downregulation of genes related with cancer [30]. Indeed SAC, caffeic acid (CA), uracil, diallyl trisulfide (DATS, as known as Allitridin), diallyl sulfide (DAS), and other garlic-derived compounds can inhibit transcription factor NF-*κ*B, a master regulator, inhibiting the transcription of several cytokine genes involved in proinflammatory responses, such as* TNF-α*, interleukin-1beta (*IL-1β*),* IL-6*,* MCP-1*, and* IL-12(p70)* [25, 48–50].

### 3.2. Phagocytosis Promotion and Macrophage Activation

The Th1 cytokine pattern is essential for controlling parasite load during the early phase of malaria infection. Feng et al. found that allicin administered to Balb/c mice postinfected with* Plasmodium yoelii* reduced parasitemia and prolonged survival due to the enhancement of proinflammatory mediators such as interferon-gamma (IFN-*γ*); additionally, allicin treatment stimulated the expansion of CD4^+^ T cells and macrophages [34]. The antimicrobial activity of allicin was demonstrated by modulation of the cytokines activating macrophages that controlled the parasitic infection.

### 3.3. Immunoglobulin Production

Modulation by means of a Th2 profile aids in the generation of an efficient humoral immune response. Washiya et al. investigated, in a mouse model, the effects of an oil-macerated garlic extract that contained Z-ajoene. The authors found that fecal IgA levels increased after 3 weeks of treatment and concluded that ajoene may have exerted an influence on B-cell stimulation or interleukin secretion [36]. Hanieh et al. proved that dietary* Allium sativum* and* Allium cepa* at low doses in white Leghorn chickens, following immunization with Newcastle Disease Virus (NDV), Sheep red blood cells (SRBC), and* Brucella abortus* (BA), enhanced anti-NDV, anti-SRBC, and anti-BA antibody production. The authors concluded that enhanced T cell proliferation with dietary garlic might has directly/indirectly enhanced B-cell proliferation and differentiation [35]. However, opposite results have been reported with garlic in the induction of antibody secretion. Jafari et al. reported that supplementing broilers with garlic do not have any beneficial effects on antibody production [51]. Therefore, more studies with garlic and its derivatives are necessary in order to clarify the mechanism implicated in immunoglobulin production.

### 3.4. Antiallergic and Allergic Properties of Garlic

An allergic reaction involves the secretion of immunoglobin E (IgE) and inflammatory mediators by immune cells. Kyo et al. found that AGE possesses antiallergic properties. In a rat basophil cell line, RBL-2H3, these authors induced histamine release with monoclonal antibodies, and after AGE administration, this significantly inhibited the antigen-specific histamine release. In addition, in a mouse model, orally administered (o.a.) AGE significantly decreased the index of immunoglobulin IgE-mediated skin reaction [37]. Zare et al. investigated the effect of intraperitoneal (i.p.) injections of AGE on an established allergic-airway inflammation murine model and observed that AGE treatment caused a significant decrease in the hallmark criteria of allergic-airway inflammation [38]. On the other hand, dietary garlic lectins have been shown to release histamine from mast cells and basophils as a result of their interaction with cell-surface IgE molecules [52]. Recently, Clement et al. isolated three immunomodulatory proteins (QR-1, QR-2, and QR-3) from raw garlic. In humans, skin prick test (SPT) using QR-1 and QR-2 on atopic and nonatopic subjects revealed that ~26% (in the case of QR-2) of atopic subjects demonstrated a positive reaction, compared with negative reactions in the case of nonatopic (normal) subjects. QR-2 induced histamine release from leukocytes to a much greater degree in the case of atopics compared with nonatopics [39]. Results noted the propensity of garlic lectins to nonspecifically activate mast cells and basophils in atopics as a result of the higher density of IgE in these patients.

### 3.5. Immunostimulatory Activities of Garlic

Fructooligosaccharides (FOS) are fructans that are naturally present in garlic. Chandrashekar et al. isolated fructans present in AGE: high molecular weight (>3.5 kDa; HF) and low molecular weight (<3 kDa; LF), which were assessed in an immunostimulatory mouse model. Both HF and LF displayed mitogenic activity and activation of macrophages including phagocytosis. These activities were comparable with those of known polysaccharide immunomodulators, such as zymosan and mannan [45]. Additionally, similar results have been obtained with immunoproteins QR-1, QR-2, and QR-3, present in garlic and identified as lectins or agglutinins [39] were previously described as ASA II and ASA I [53], and their mitogenic and comitogenic properties were confirmed as comparable with potent mitogenic lectins ConA and PHA. On the other hand, it is well known that fructans selectively stimulate some beneficial bacteria in colon, modulating different immune responses [54, 55].

Despite increasing evidence, the different components in garlic responsible for effective immune stimulation or inhibition are not known conclusively, and it is likely that several components are responsible for its immunopharmacological mechanisms. Therefore, further research on garlic fructans may cast light on the underlying mechanisms of immunomodulation and should aid in identifying potential uses of garlic fructans in various therapeutic applications [45].

## 4. Effects of Garlic Compounds/Extracts on Cells of the Immune System

Different studies have shown that garlic compounds are able to perform antiapoptotic [56], antiparasitic [11], proapoptotic, anticancerigenic [57], and immunomodulatory [58, 59] effects on different cells.

It was observed in a murine macrophages cell line infected with* Leishmania* that AGE induced IL-12 production [11] and, in addition, INF-*γ* and inducible nitric oxide synthase (iNOS) were overexpressed [12]. However, in peripheral blood monocytes, AGE upregulated IL-10 and decreased IL-12 production [60], which might cause downregulation of proinflammatory cytokines TNF-*α*, IL-6, INF-*γ*, and IL-2 by T cells and it acts as negative feedback in the signaling proinflammatory response [60–62]. Additionally, DADS decrease NO production, proinflammatory cytokines, and protein expression in a mouse leukaemic monocyte/macrophage cell line [63]. Therefore, garlic compounds could act as immunomodulatory agents on the macrophages response.

Other studies conducted in mice have been shown that DATS can enhance the antiviral immune response to murine cytomegalovirus (MCMV) [64], by blocking Treg* in vivo* in chronic MCMV infection [65]. Additionally, the protein fraction of fresh garlic stimulates the peripheral blood T-lymphocyte proliferation and increases CD8^+^ subpopulation in treated animals, causing an increase in delayed-type hypersensitivity responses, promoting an efficient cellular response [66]. However, these studies did not assess the cytokine profile, which could provide more information about the immunomodulatory role of different garlic protein subfractions.

It has been documented that garlic or its compounds induce a variety of immunomodulatory activities in leukocyte cytokine production. In Th1 cells, inflammatory cytokine production is reduced significantly in the presence of garlic extract and/or its compounds, revealing a potential therapeutic use in inflammatory conditions such as inflammatory bowel disease (IBD) [60] and malaria [34]. However, it is also known that garlic oil shifts the Th1-Th2 balance toward the Th2 type [21].

Furthermore, garlic derivatives exert both stimulatory [1] and inhibitory effects on whole blood cultures of monocytes and lymphocyte proliferation and LPS-induced TNF-*α* generation through IL-10 production, which controls proinflammatory cytokines [60]. Moreover, other compounds, such as allicin, exert negative effects on human T-cell migration through fibronectin by downregulating actin reorganization [67]. Even more so, protein fraction 4, isolated from AGE, enhances the cytotoxic activity of human peripheral blood lymphocytes (HPBL) in synergy with IL-2 and independently from INF-*γ* or TNF-*α* [68].

Finally, the *γδ*-T population, as a unique type of T cell that recognizes and responds to pathogen-associated molecular patterns (PAMP), increases its proliferation by AGE supplementation in healthy humans [69]. Taken together, these data strongly suggest that garlic compounds and its derivatives are involved in the cellular immune response, acting as immunoregulators; however, more studies are needed to clarify its use in immunotherapy.

Proteins isolated from garlic modulate NK cell line activity in the mesenteric lymph node of mouse [70], while AGE modulates the number and the activity of NK cells in patients with various advanced cancers [71] and also increases NK activities against different cancerous cell lines [72]. Moreover, in healthy subjects, AGE increases the NK cell population [69]. Therefore, garlic acts as a proliferation inductor for this cell type.

Mature dendritic cells (DC) can activate naïve lymphocytes and play a critical role in the induction of primary immune response [73]. Allicin treatment could promote the maturation of DC by increasing the expression of costimulatory molecules such as CD40, yielding an enhancement of the proinflammatory immune response in a rodent malaria model [34]. However, it was not possible to establish whether the 14 kDa protein isolated from AGE induced mouse DC* in vitro* maturation by an increase in the expression of the CD40 molecule in DC [74]. Consequently, future studies are needed to determine the effect of garlic on DC.

Garlic allergens have been reported as causing hypersensitivity reactions in both patients and animal models [75–77], such as dermatitis [78], rhinoconjunctivitis, asthma [79–81], urticaria [82], and anaphylaxis [83] after ingestion of garlic. This can be due to cross-reactivity in patients with oral allergy [82]. Recently, it was demonstrated that a 56-kDa protein of alliin lyase is the major IgE-binding protein in patients allergic to garlic. Alliin lyase contains a carbohydrate with free terminal *α*-D-glucopyranoside or *α*-mannopyranoside residues, thought to bind human IgE in subjects with allergy and to lead to cross-reactivity [77]. Additionally, three protein components from raw garlic displayed hemagglutination and mannose-binding activities; one of these induces histamine release from human leukocytes [39]; likewise, garlic lectins are able to evoke immunogenicity [39, 84]. However, the molecular basis of the interaction between food allergens and the immune system is not clear.

## 5. Role of Garlic Compounds in Inflammatory Disorders

Numerous research works have shown the immunomodulatory and immunotherapeutic potentials of AGE as a whole, including free radical-mediated anti-inflammatory, anticancer, and antiangiogenic effects, as well as improving hyperglycemia and dyslipidemia, cardiovascular diseases, infectious diseases, autoimmune diseases, and allergy, which have been shown in both animal models and cell lines [28, 85–87]. It is known that the aqueous garlic extract exerts antioxidant action by scavenging reactive oxygen species (ROS) and enhancing cellular antioxidant enzymes such as superoxide dismutase, catalase, and glutathione peroxidase. In addition, garlic represents an important source of antioxidants due to phytochemicals such as DAS and SAMC [28, 88].

### 5.1. Metabolic Syndrome

The metabolic syndrome is a cluster of abnormalities including hypertension, insulin resistance, hyperlipidemia, glucose intolerance, and abdominal obesity. This syndrome frequently precedes type 2 diabetes and atherosclerosis [89]. The role of garlic has been studied in some of these pathologies, and their effects on the immune system components associated with the proinflammatory state of metabolic syndrome include modulation of oxidative stress (OS), proapoptotic signal pathways, inflammatory mediators, and cellular activities.

#### 5.1.1. Cardiovascular Disorders

Cardiovascular diseases (CVD) continue to accelerate globally and remain the largest cause of deaths worldwide. CVD include diseases of the heart, vascular diseases of the brain, and diseases of blood vessels [90]. Plasma markers of inflammation have also been evaluated as potential tools for prediction of the risk of coronary events. Among these are markers of systemic inflammation, such as high-sensitivity C-reactive protein (CRP), and acute-phase protein [91], serum amyloid A, cytokines such as IL-6, and adhesion molecules such as soluble intercellular adhesion molecule type 1 (ICAM-1) [92, 93] and vascular cell adhesion molecule-1 (VCAM-1) [94]. The participation of ROS and the activity of endothelial nitric oxide synthase (eNOS) in vascular alterations [95, 96] have been reported.

Several studies* in vitro,* have confirmed the cardioprotective effects of garlic on primary cultured cardiac myocytes, fibroblasts, and endothelial cells, by reducing the production of ROS and blocking ROS-dependent extracellular signal-regulated kinase (ERK)1/2, JNK1/2, AKT, NF-*κ*B, and SMADS signaling [25, 97, 98]. However, garlic powder exerts no detectable effects on CRP, TNF-*α*, ICAM-1, lipid concentrations, and risk markers for inflammatory processes associated with subjects with atherosclerosis and CVD [99]; additionally, AGE does not change plasma cholesterol level or ICAM-1 expression in a rabbit model of atherosclerosis [100]. However, studies do not reflect the entire population-at-risk for atherosclerosis and cardiovascular diseases because these studies underwent adverse events in disease course (e.g., significant numbers of subjects withdrew from the study). In contrast, recent data showed that long-term administration of aqueous garlic was capable of attenuating VCAM-1 expression in fructose-fed rats. Therefore, garlic compounds reduce vascular inflammation [25, 94].

Atherosclerosis is recognized as a complex disease characterized by an excessive inflammatory, fibrofatty, and proliferative response to damage in the vascular endothelium and involving several cell types, particularly smooth muscle cells, monocyte-derived macrophages, T-lymphocytes, and platelets [101, 102]. Clinical reports have revealed the potential benefits of garlic as a modulator of multiple cardiovascular features through lowering low-density lipoproteins (LDL) and blood pressure [103–106], reducing platelet aggregation and adhesion, preventing LDL oxidation, and reducing the progression of atherosclerosis [100, 107–109]. However, it is known that some garlic compounds, such as DADS and allyl mercaptan, did not inhibit the transcriptional activity of factor NF-*κ*B employing human umbilical endothelial cells, suggesting that they play a pivotal role in atherogenesis by regulating the expression of proinflammatory genes and of NF-*κ*B-regulated genes, suggesting that NF-*κ*B is not the major target of DADS and allyl mercaptan. Accordingly, there are differential effects among different organosulfur compounds of garlic [110]; thus, more research is needed to discriminate the beneficial effects accurately and to ascribe these to specific garlic compounds.

#### 5.1.2. Obesity

Obesity is associated with low-grade chronic inflammation characterized by abnormal cytokine production, increased acute-phase reactants, and other mediators in response to excess nutrients in metabolic cells [111]. Activation of a network of inflammatory signaling pathways in the cell eventually causes the activation of specialized immune cells and leads to an unresolved inflammatory response within the tissue [112]. Thus, macrophage [113], mast-cell [114], and NK-cell [115] infiltration is present in obese adipose tissue, which participates in the inflammatory changes in obesity and contributes to insulin resistance [113].

Garlic 1,2-vinyldithiin reduces the secretion of IL-6 and MCP-1, -2 in human preadipocytes treated with macrophage factors. Both molecules are associated with inflammation and the metabolic complications of obesity [116]. Recently, our group demonstrated that alliin prevents the increase of genes and proteins related with the proinflammatory state induced by LPS in 3T3-L1 adipocytes, through the toll-like receptor-4 (TLR-4) pathway and possibly, by regulating ERK1/2 activity [30].

#### 5.1.3. Ulcerogastric Pathologies

In gastric pathophysiology, T and B cells are clearly involved. OS causes damage to lipids, proteins, and DNA [28, 117]. In this respect, garlic has been studied as a gastroprotective agent. AGE capsules have been capable of resolving indomethacin-induced OS in gastric tissue through a reduction of TNF-*α* and malondialdehyde levels and reduction of myeloperoxidase activity, as well as increasing total glutathione, superoxide dismutase, and catalase activities in animal model [28]. Additionally, garlic oil administered to rats prior to ethanol administration induced a decrease in ulcer index and lipid peroxidation and ameliorated the decrease in antioxidant enzyme levels caused by ethanol [118]. Therefore, garlic can be considered an excellent preventive and protective agent to reduce gastric pathologies.

The anti-inflammatory effect of the garlic extract by IL-10 deregulation and the reduction of IL-12 production in Inflammatory bowel disease (IBD) prevents IL-12 from binding to its receptor on T and NK cells, causing inhibition of the production of IFN-*γ* [60].

#### 5.1.4. Cancer

Numerous health benefits have been ascribed to organosulfur compounds, including its immunomodulatory properties in cancer [9, 119–121]. A report in the literature noted an association between garlic consumption and decreased incidence of distal colon cancer in women in a cohort study [120]. It has been proposed that allicin presents antitumor activity* in situ* [122]. More specifically, cultured Ehrlich ascites carcinoma (EAC) cells treated with tamoxifen and supplemented with allicin resulted in cytotoxic damage markers and a decrease in TNF-*α* levels [121]. Hence, a beneficial role of allicin is suggested as an adjuvant to tamoxifen treatment in cancer.

Recent work also showed that SAC and DATS cause inhibition of PI3K/Akt, JNK apoptotic pathways in human ovarian, and T24 human bladder cancer cells [57, 123]. Even more allicin induces apoptosis through JNK pathway activation and mitochondrial Bax translocation in cells human ovarian cell line SKOV3 [124]. Other studies have demonstrated the role of protein fractions from garlic bulbs in tumor growth and intratumor-infiltrated T lymphocytes in mice transplanted with mammary tumor cells [66], as well as a significant decrease in the size of mouse mammary tumor [70] and complete suppression of growth of Human erythroleukemia cell line HEL in a dose-dependent manner [125]. Recently, our group evidenced that alliin treatment of 3T3-cell-derived adipocytes is capable of downregulating several cancer-related genes [30]. Thus, garlic compounds could significantly affect the tumor development, thorough, at least, their antiproliferative action.

Other groups have shown that fraction 4 of AGE, combined with IL-2 administration, could be employed in tumor immunotherapy, because these increase the cytotoxicity of T-cell lineage [68], and it has been proposed that the sulfhydryl-group hydrophobic portion of proteins, as well as estrogen receptors with cysteine residues in hormone-binding, could be target of inhibition from organosulfur compounds of garlic, (e.g., allyl sulfides). This may be of greater benefit in the prevention of hormone-responsive carcinogenesis [125]. Thus, while total sulfur may be comparable, marked differences in specific organosulfur components likely exist among the preparations studied [126], which strongly suggest that the antitumor effect of allyl sulfur compounds may be related with both their anti-inflammatory and their immunostimulatory properties.

## 6. Concluding Remarks

Garlic is one of the most employed seasonings for cooking. In addition to its use as a food additive, garlic has been long used in traditional medicine with protective and curative purposes. At present, the trend toward the use of natural remedies with fewer side effects has given rise to garlic consumption as an alternative therapy for diseases such as cardiovascular diseases, cancer, and microbial infections. Different dietary garlic formulations, such as powder (tablets), garlic oil (capsules), and aged garlic extracts (tablets, capsules, and liquids), have been incorporated into the globally increased market of garlic bioactive compounds. However, the variety of manufacturing processes of garlic comprises important issues when choosing a garlic supplement, due to that these processes can markedly influence the composition of the garlic product and thus its biological effects.

Garlic as an herbal medicine or its different bioactive molecules and formulations have been extensively probed in* in vitro/in vivo* animal models to examine its anti-inflammatory and immunomodulatory properties. One of the main mechanisms observed is through modulation of cytokine profiles and, on the other hand, direct stimulation of immune cells. Although there is sufficient scientific evidence on the beneficial effects of garlic as therapy under different pathological conditions in animal models, human clinical studies are scarce and methodologically weak, with short duration and a reduced number of patients. Therefore, it is mandatory to establish general criteria to finally probe the variety of nutritional and health-promoting properties of garlic.

## Figures and Tables

**Table 1:** Biological effects of different types of garlic preparations and extracts.

Preparations/extract	Effects	References
Dehydrated garlic powder/slices/crushed	Diminish serum cholesterol	[8]

Aqueous extracts	Antibacterial	[9, 10]
	Antiparasitic	[11]
	Modify immune response	[12]
	Lipid metabolism	[13]
	Cardiovascular-protective effects	[14, 15]

Oil extracts	Antibacterial	[16–19]
	Acaricidal	[20]
	Modify Immune response	[21, 22]

Chloroform extract	Inhibiting ROS formation and attenuating the activities of adhesion molecules	[23]

Hexane extract	Cytotoxic	[24]
	Modify immune response	[25]

AGE	Antioxidant	[26, 27]
	ROS scavenger and anti-inflammatory	[28]
	Inhibits development of preneoplastic lesions	[29]

**Table 2:** Immunoregulatory properties of garlic.

Immunoregulatory mechanism	Model/pathology involved	Garlic preparation (dose)	Immunoparameters evaluated	Conclusions	References
	3T3-L1 adipocytes stimulated with LPS/*in vitro* model of inflamed adipose tissue.	Cell incubation with alliin for 24 h (100 *μ*mol/L).	Proinflammatory cytokines and adipocytokines IL-6, TNF-*α*, MCP-1, and adiponectin.	Alliin is capable of suppressing LPS inflammatory signals by generating an anti-inflammatory gene expression and prevented the increase in expression of proinflammatory cytokines IL-6 and MCP-1.	[30]
	Male Wistar rats/inflammation.	Gavage with garlic oil (10–200 mg/kg).	Cellularity of cervical lymph nodes. Production of Th1 cytokines IL-2 and IFN-*γ* and Th2-type cytokines IL-4 and IL-10.	Garlic oil enhances and shifts toward Th1-type response at low doses. It promotes an anti-inflammatory environment at high doses by shifting Th1-Th2 balance toward the Th2 type.	[21]
Modulating cytokine secretion	Preeclamptic placental explant tissue stimulated with LPS.	Garlic extract (10, 100, 500, and 1,000 *μ*g/mL).	Cytokine levels of TNF-*α*, IL-6, IL-10, and sTRAIL.	Garlic at lower doses possesses an immunomodulatory effect on normal placenta by increasing production of IL-10 and in preeclamptic explants reduces production of inflammatory cytokines such as IL-6 and TNF-*α*. At higher doses, overall effect is one of cytokine synthesis inhibition and stimulation of sTRAIL production.	[31]
	Whole blood stimulated with LPS and human embryonic kidney cell line 293 (HEK293).	Garlic powder extracts (10 g/L), DADS (100 mol/L), and allicin (100 mol/L).	Cytokine levels of TNF-*α*, IL-1*β*, IL-10, and NF-*κΒ* activity.	Garlic compounds modulate inflammatory cytokines, leading to overall reduction of NF-*κ*B activity.	[32]
	*In vitro*: peritoneal macrophage-mediated antitumoral activity.	Allicin (1, 10, and 100 ng/mL) for 20 h.	Cytotoxicity and phagocytosis assay. Nitrite and hydrogen peroxide production. Production of cytokines TNF-*α*, IL-1, and IL-6.	Allicin increases macrophage production of TNF-*α* and nitric oxide (NO) in a dose-dependent manner.	[33]

Phagocytosis and cell activation	Balb/c mice infected with *Plasmodium yoelii*/Malaria.	Allicin orally applied 3 or 9 mg/kg/day on days 0–2 (PI).	Pro- and anti-inflammatory cytokines IFN-*γ*, TNF-*α*, IL-12p70, IL-4, and IL-10.	Allicin reduced parasitemia and prolonged survival due to improved host immune responses. Enhancement of proinflammatory mediators IFN-*γ*, TNF-*α*, and IL-12p70. No changes in anti-inflammatory cytokines IL-4 and IL-10.	[34]
	*In vitro *assays: neutrophil-like cells (HL-60 cell line).	Garlic oil (1 *μ*g/mL < 10 *μ*g/mL) for 60 min.	Chemotactic responsiveness and motility of neutrophil-like cells.	Average migration speed of cells reduced after being treated with garlic oil, thereby resulting in anti-inflammatory activities through inhibition of assembly and disassembly of cytoskeleton inside the cell.	[22]

Activation of humoral immune response and synthesis of Ig	*In vivo *assays, white Leghorn chickens/viral and bacterial infection.	Dietary alliums: *Allium sativum *(G) and *Allium cepa* (O) (low doses: 10 g/kg (GL and OL) or high doses 30 g/kg (GH and OH)).	Antibodies, lymphocyte proliferation, and ratios of CD4^+^ : CD8^+^ and CD4^−^ : CD8^−^ lymphocytes.	GL and OL enhanced anti-NDV, anti-SRBC, and anti-BA antibody productions. Only GL- and GH had a comitogenic effect on splenocytes and thymocytes. Reduction in CD4^+^ and increase in CD4^−^ : CD8^−^ lymphocyte ratios were observed with GH or OH.	[35]
	Mouse mucosal.	OMG containing 1,500 mg/g of ajoene.	IgA production in feces or colon tissue.	Intestinal IgA level was increased by ajoene; thus, ajoene may have influenced B-cell stimulation or interleukin secretion.	[36]

Antiallergic response	*In vitro *assays: RBL-2H3 induced by (TNP) monoclonal antibody and the TNP (BSA-related) hapten carrier complex/allergic reactions. *In vivo *assays: Balb/c male mice i.v. administered anti-TNP IgE antibody and subsequent picryl chloride painting on the ear/allergic reactions.	AGE incubation (1.25, 2.5, and 5.0 g/100 g). AGE orally applied (10 mL/kg).	Histamine release by basophils. Ear swelling used as an index of immunoglobulin IgE-mediated skin reaction.	AGE significantly inhibited antigen- specific histamine release and decreased ear swelling. AGE may directly and/or indirectly modify functions of mast cells, basophils, and activated T lymphocytes, which play a leading role in allergic cascade reactions.	[37]
	Balb/c mouse allergic-airway inflammation/asthma.	3 IP injections of 14 kD fraction of AGE (20 mg/kg).	Percentages of lavage eosinophils. Mucus-producing goblet cells in airways. Perivascular and peribronchial inflammatory grades.	14 kD fraction of AGE is able to reduce allergic-airway inflammation hallmarks in murine model accompanied by increase in IFN-*γ*-level bronchoalveolar lavage.	[38]

Mitogenic stimulator	*In vitro *assays on immune cells/immunomodulation.	Garlic protein fractions: QR-1, QR-2, and QR-3.	Proliferation index in murine splenocytes/thymocytes and human PBL.	All three proteins exhibited mitogenic activity toward human PBL and murine splenocytes/thymocytes. Mitogenicity of QR-2 was the highest among the three immunomodulatory proteins.	[39]
	*In vitro *assays on PBMC and PMN incubated with or without 10 ng/mL of LPS.	Alliin (1 and 3.0 mg/mL).	Cytokine concentration: IL-1*β*, IL-6, TNF-*α*, and IL-2. Superoxide anion production. Phagocytosis.	Alliin induces PWM-cell proliferation, spontaneous production of IL-1*β*, as well as an increase in number of phagocyting cells and engulfed latex particles. Alliin causes decrease in mitogenic function of ConA.	[1]

Anti-inflammatory and antioxidant effects	Male albino rats (*Rattus norvegicus*)*/*gastric inflammation.	AGE orally (100–200 mg/kg).	Macroscopic appearance of gastric mucosa. Microbial count. Levels of TNF-*α*, SOD, CAT, and MPO enzyme activity.	Gastroprotective mechanism of AGE on gastric damage induced by Indomethacin through its anti-inflammatory actions and its antioxidant properties.	[28]

Aged garlic extract (AGE); malondialdehyde (MDA); myeloperoxidase (MPO); total glutathione (tGSH); superoxide dismutase (SOD); catalase (CAT); peripheral blood lymphocytes (PBL); peripheral blood mononuclear (PBMC); polymorphonuclear (PMN); pokeweed mitogen (PWM); tumor necrosis factor- (TNF-) related apoptosis-inducing ligand/Apo-2L (sTRAIL).
